# Antibiotic use and financial impact of a comprehensive beta-lactam allergy management program

**DOI:** 10.1017/ash.2025.36

**Published:** 2025-04-10

**Authors:** Karan Raja, Lakhini Vyas, Susan Morrison, Donald Beggs, Mitesh Patel, Mona Philips

**Affiliations:** 1 Pharmacy Department, Clara Maass Medical Center, Belleville, NJ, USA; 2 Rutgers, The State University of New Jersey, New Brunswick, NJ, USA; 3 Medicine Department, Clara Maass Medical Center, Belleville, NJ, USA

## Abstract

**Objective:**

A multidisciplinary beta-lactam allergy management program was implemented at our community medical center to facilitate allergy documentation, conduct penicillin skin testing (PST), and decrease non-beta-lactam (NBL) use. This study measures PST-associated antibiotic use and financial outcomes.

**Design:**

Cohort study.

**Setting:**

Non-teaching, urban, community medical center within a multi-hospital health system.

**Patients:**

Adult inpatients who underwent PST and received antibiotic therapy during a 5-year period at our facility.

**Methods:**

Demographics, allergies, laboratory results, PST outcome, and antimicrobial regimens were assessed. Actual NBL days of therapy (DOT) were collected from the electronic medical record. NBL DOT that patients would have received without PST were modeled by forecasting the original regimen to end of inpatient treatment. Difference between actual and forecasted DOT was deemed DOT avoided (DOT-A) for each consecutively enrolled patient. The financial analysis evaluated cumulative NBL cost avoided. PST outcomes and average time from antibiotic initiation to PST were assessed.

**Results:**

The study included 600 patients who underwent PST an average of 3.7 days into treatment. The most common indication was acute bacterial skin and skin structure infections (23.9%). PST results were negative in 98% of patients. NBL DOT-A was 944.8/1000DT (8.8 DOT-A per intervention) accounting for an estimated cost savings of $206,500 ($344.10 per intervention), driven primarily by aztreonam avoidance.

**Conclusions:**

This study highlights significant avoidance of NBL DOT in one of the largest identified cohort of inpatients undergoing PST. Associated cost avoidance contributes to the sustainability and longevity of the allergy management program.

## Introduction

Beta-lactam class antibiotics are among the safest and most efficacious agents used in treating various bacterial infections.^1–4^ However, beta-lactams account for the majority of inpatients’ self-reported medication allergies in the United States.^1^ Published literature confirms that a majority of patients do not have an IgE-mediated reaction upon skin testing and can be safely transitioned to an appropriate, targeted beta-lactam.^1,2^ Additionally, a significant number of patients with reported anaphylaxis to penicillins may lose their sensitivity over time.^2^

Allergy misclassification increases use of broad-spectrum, alternative antibiotics, potentiates antimicrobial resistance, and increases cost of care.^1–3,5–8^ This can heighten the risk of suboptimal infection-related outcomes, prolonged hospital stays, and readmission.^1,6,9–11^ NBL prescribing likely stems from allergy documentation rather than microbiologic criteria in patients with a documented beta-lactam allergy.^1,6^ Therefore, clarifying antibiotic allergy status is beneficial in both immediate and long-term antimicrobial stewardship efforts. Previous studies have also described an increase in direct antimicrobial treatment cost in patients with a documented penicillin allergy.^12,13^ Trubiano and colleagues compared oncology patients with and without an antimicrobial allergy label (primarily beta-lactams, 65%).^11^ Those with an allergy label experienced increased overall antibiotic use per admission, increased fluoroquinolone use, longer antibiotic courses, and higher readmission rates.

The Infectious Diseases Society of America and American Academy of Allergy, Asthma, and Immunology advocate for avoiding non-beta-lactam (NBL) antibiotic overuse without thorough allergy evaluations.^14,15^ Comprehensive allergy management programs should include detailed allergy history-taking, risk stratification, and penicillin skin testing (PST) or direct drug challenge if needed.^3,5^ A thorough allergy history is often sufficient to rule out significant allergy and allows for beta-lactam use.^5^ PST additionally offers a >95% negative predictive value for ruling out IgE-mediated hypersensitivities and is an instrumental component of antimicrobial stewardship programs.^2,3,16–20^

Studies evaluating PST programs have demonstrated a reduction in NBL antibiotic days of therapy (DOT), length of hospital stay (LOS), hospital readmissions, and cost of care, by avoiding costly alternatives or combination therapy.^1,2,5,7,8,21^ Heil and colleagues found a reduction in aztreonam use from 3.4 to 1.9 DOT/1000 PD after PST implementation.^22^ This corresponded to approximately $26,000 in savings per year. However, there are limited studies assessing the financial and NBL use impact of a comprehensive allergy management program in such a large patient population, particularly in non-teaching, community medical centers without medical residents or ID fellows.

A multidisciplinary beta-lactam allergy management program was implemented at our institution to facilitate complete allergy documentation and increase use of targeted beta-lactams. The objective of the current study is to evaluate NBL utilization and financial outcomes associated with this program. To our knowledge, this study presents outcomes in one of the largest published inpatient PST cohorts.

## Methods

This study was conducted at a non-teaching, urban, community medical center with a multidisciplinary allergy management program established in April 2019. As part of this existing program, pharmacists, infectious disease physicians, and an allergy and immunology physician identified inpatients with a beta-lactam allergy and collected detailed medication allergy histories. Patients were risk stratified as high, moderate, or low risk of IgE-mediated allergy based on the reaction history. Direct beta-lactam drug challenge was recommended for patients with low-risk histories. Moderate-risk patients were recommended to be further evaluated with PST. Beta-lactam avoidance was recommended in high-risk patients. For de-labeled patients, the pharmacist and allergist collaborated with hospitalists or ID consultants to optimize antimicrobial therapy and increase use of beta-lactams, as appropriate. We previously published details of this program.^23^

The electronic medical record (EMR) was queried for all inpatients who underwent PST at our institution from program initiation (April 11, 2019). Patients were excluded if not on inpatient antibiotic therapy or were under 18 years old. Patients were consecutively screened and enrolled. Patient demographics, allergy information, antimicrobial indications, laboratory and culture results, PST results, and antimicrobial therapy regimens were retrieved from the EMR. Patient’s actual antibiotic DOT was collected from the EMR. “Actual NBL DOT” included total NBL DOT that the patients received throughout the treatment course, before *and* after undergoing PST. We assumed that if no PST had been conducted, patients’ antibiotic therapy before PST would have remained unchanged for the remainder of the treatment course, termed “forecasted NBL DOT.” Treatment course was defined as antibiotic therapy for the current indication(s) and was censored at hospital discharge if needed. The difference between actual and forecasted DOT was deemed DOT avoided (DOT-A) for each patient. Although all antibiotic regimens were evaluated, this study focused on decreasing NBL use. Outcomes evaluated include total NBL DOT-A normalized per PST intervention and per 1000 days of inpatient antibiotic treatment (1000DT). The financial analysis evaluated the cumulative NBL cost avoided normalized per PST intervention and per 1000DT. The cost per day was calculated by multiplying the defined daily dose (DDD) of each antibiotic by the wholesale acquisition cost (WAC), as of April 2024, in USD. The DDD and WAC were used to allow for greater generalizability in various practice settings. Additional endpoints evaluated were PST outcomes and the average time into antimicrobial therapy that PST was conducted to uniquely frame the impact on our results. Outcomes were assessed with descriptive statistics, including measures of central tendency and dispersion.

## Results

**Figure 1.**
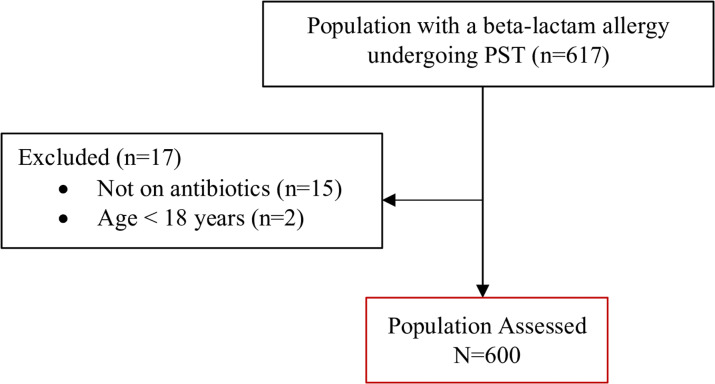
Patient CONSORT diagram.

Between April 1, 2019 and July 8, 2024, there were 617 inpatients with a beta-lactam allergy who underwent PST at our institution. After excluding those without inpatient antimicrobial therapy or under 18 years old, 600 patients were included for study analysis (see Figure 1). Patients were an average of 67.4 ± 16.8 years old and a majority were female (69%). The average hospital length of stay was 11.2 ± 8.5 days and average duration of antibiotic therapy was 9.2 ± 7 days. Patients underwent PST an average of 3.7 ± 3.6 days into antimicrobial treatment.

There were 613 distinct beta-lactam allergies and 669 antimicrobial indications in the study population, as some patients had more than one drug allergen or infection source documented. There were 554 patients with a penicillin class allergy (90.4%), 54 patients with a cephalosporin class allergy (8.8%), and 5 patients with a carbapenem class allergy (0.8%). Antimicrobial indications included acute bacterial skin and skin structure infections (23.9%), urinary tract infection (23.3%), pulmonary infection (21.5%), intra-abdominal infection (13.9%), and other infections (17.4%). Upon PST, 588 patients (98%) tested negative, 8 (1.3%) tested positive, and 4 (0.7%) were indeterminate due to recent antihistamine administration. There were two instances of antimicrobial intolerance after administration of optimized therapy after negative PST. All baseline characteristics are shown in Table 1.

**Table 1.** Baseline characteristics

Characteristic	
Age – year, mean (SD)	67.4 (16.8)
Female sex – no (%)	414 (69)
Length of stay – days, mean (SD)	11.2 (8.5)
Duration of antibiotic therapy – days, mean (SD)	9.2 (7.0)
Antibiotic Allergen Class – no (%)	*Total Allergens = 613*
Penicillin	554 (90.4)
Cephalosporin	54 (8.8)
Carbapenem	5 (0.8)
Antibiotic Indication – no (%)	*Total Infections = 669*
Skin and soft tissue	160 (23.9)
Urinary tract	156 (23.3)
Pulmonary	144 (21.5)
Intra-abdominal	93 (13.9)
Other	116 (17.4)

Note. Some patients had multiple allergens documented and were treated for multiple infections; therefore, the total number of allergens and infections is greater than the total number of patients.

**Figure 2.**
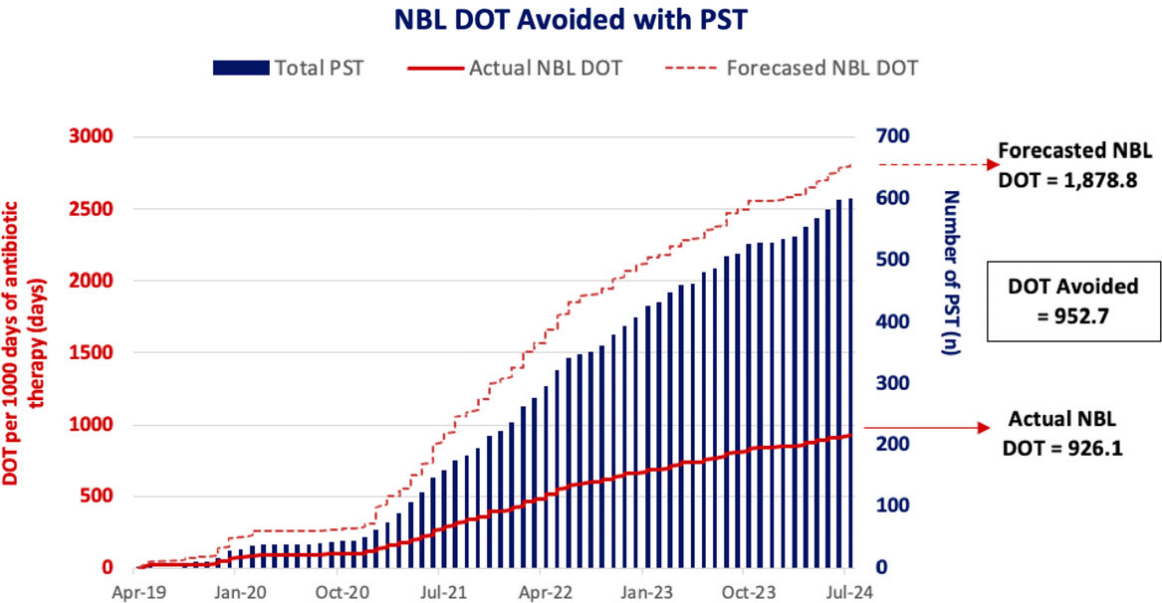
Non-beta-lactam days of therapy per 1000 days of antibiotic therapy. Abbreviations: NBL, non-beta-lactam; DOT, days of therapy.

Amongst the 600 patients evaluated, total NBL DOT-A was 5,249 days and total associated cost savings were $206,458. This equated to NBL DOT-A of 952.7 per 1000DT or an average of 8.8 ± 12.3 DOT-A per PST intervention (see Figure 2). Total cost savings was $37,469.69 per 1000DT or an average of $344.10 ± $554.38 per PST intervention, driven primarily by aztreonam avoidance. NBL DOT and cost outcomes are shown in Table 2. Cost savings for each NBL agent is shown in Table 3.

**Table 2.** Cost of NBL therapy

	Forecasted	Actual	Avoided
NBL DOT (days)	10,352	5,103	5,249
NBL DOT per 1000DT	1,878.8 ± 3.24	926.1 ± 1.65	952.7 ± 2.2
NBL DOT per intervention	17.3 ± 0.03	8.50 ± 0.02	8.8 ± 0.02
Total NBL Cost	$380,609	$174,151	$206,458
NBL cost per 1000DT	$69,076.04 ± $173.30	$31,606.35 ± $106.09	$37,469.69 ± $100.56
NBL cost per intervention	$634.35 ± $1.59	$290.25 ± $0.97	$344.10 ± $554.38

Abbreviations: NBL, non-beta-lactam; DOT, days of therapy; 1000DT, 1000 days of antibiotic therapy.

**Table 3.** Non-beta-lactam cost avoided

	Total DOT Avoided	Cost per day^a^	Cost Avoided ($)
Aztreonam	1,735	$92.00	$159,620
Vancomycin	893	$16.00	$14,288
Clindamycin	387	$33.00	$12,771
Linezolid	223	$24.00	$5,352
Doxycycline	240	$16.00	$3,840
OTHER	1,771		$10,587
Total	**5,249**		**$206,458**

a Cost per day is calculated based on WHO DDD × WAC cost in USD (as of April 2024).

## Discussion

This study evaluated a large inpatient PST cohort that demonstrated significant NBL DOT and cost avoidance per PST intervention, supporting program financial viability and sustainability. PST benefits likely extend beyond these study outcomes, contributing to reduced morbidity, mortality, and hospital LOS with targeted antimicrobial therapy^25^, likely secondary to greater effectiveness and/or lower adverse event rates.^24,25^ Additionally, optimal inpatient management and stewardship positively impact outpatient antibiotic use and costs.^25,26^

There were 8.8 ± 12.3 NBL DOT-A per PST intervention or an average of 952.7 NBL DOT-A per 1000DT. Chen and colleagues evaluated 228 patients who underwent PST in a pharmacist-allergist collaboratively managed program, where 504 inpatient days (2.2 DOT-A per PST intervention) and 648 outpatient days of alternative antibiotic therapy were avoided.^26^ Ramsey and colleagues described an PST program that avoided 982 days of combined inpatient and outpatient second-line antibiotic therapy.^25^ Differences in DOT-A rate may be attributable to time into therapy PST was conducted, evaluation of inpatient-only compared to combined inpatient and outpatient data and baseline NBL prescribing rates. The significant avoidance of NBL DOT after PST intervention contributed to the total cost savings of $206,458 amongst the 600 patients in the 5-year study period. This equates to $37,469.69 per 1000DT and an average of $344.10 ± $554.38 per PST intervention. Other studies have identified similar results with an estimated cost savings of $225 to $350 per patient undergoing PST.^1,19,27,28^

In our study, the expected NBL cost decreased by 54.2% after PST, from forecasted $380,609 to actual cost of $174,151 (see Table 2). Macy and colleagues evaluated 236 patients and found the average antibiotic cost per patient fell 30.6% from $71.17 to $49.63 if the subject had a negative PST.^9^ A PST program in North Carolina demonstrated an estimated cost savings of $82,000 in patients receiving antibiotic therapy changes as a result of PST.^2^

The cost savings in this study are largely driven by aztreonam avoidance. Staicu and colleagues evaluated aztreonam use after PST program implementation in 178 patients.^29^ They found a significant reduction in aztreonam DOT from 9.5 to 4.4 DOT/1000 patient days with an associated $60,000–$100,000 annualized projected cost avoidance compared to other first-line agents. Heil and colleagues evaluated 90 patients and conducted PST on 76. They found a reduction in aztreonam use from 3.4 to 1.9 DOT/1000PD after PST implementation, corresponding to approximately $26,000 in savings per year.^22^ In our study of 600 patients undergoing PST, a total of 1,735 days of aztreonam therapy were avoided, which accounted for a cost savings of $159,620 for aztreonam alone during the study period. The total cost avoided for NBL agents was $206,458, making aztreonam responsible for over 75% of total cost savings.

A strength of the study is its strong internal validity, ensured by a consistent team managing the program, which limited practice variability. Furthermore, the study reflects a pragmatic view of program outcomes, as earlier identification, assessment, and intervention of candidates could magnify study results. Treatment durations were measured observationally, reflecting true clinical practice and thereby enhancing the external validity of findings. The rate of negative PST in previous studies has ranged from 94 to 100 percent, similar to the 98% of patients who tested negative upon PST in our study.^25,30–34^ Patients underwent PST on average 3.7 days into antibiotic treatment. Some patients had antibiotics discontinued or were discharged on the day of PST. The study period also includes the peak of the COVID-19 pandemic, which likely limited program workflow due to strict infection control measures. Additionally, some patients did not have changes to their NBL antimicrobial if it was considered drug of choice.

Cost savings were derived from NBL DOT-A during the acute treatment course, not including beta-lactam therapy costs or program implementation expenses. However, while not directly measured in this study, PST programs are likely cost-effective overall as previous studies estimate a cost of $256 [2020 USD] per PST, though variability exists.^35,36^ Additionally, patients with antimicrobial allergy labels face higher healthcare burdens, including increased risk of hospital-acquired infections (eg *Clostridioides difficile* infection), adverse drug reactions, and hospital readmissions.^12,37,38^ Therefore, cost-effectiveness of PST encompasses factors beyond direct drug costs, demonstrates sustainability, and offers enduring value. Larger studies are needed to assess long-term clinical and economic outcomes and the impact of allergy re-labeling after PST. Cost evaluation was based on WAC of each drug as of April 2024 and DDD as per the World Health Organization. Actual cost savings may vary as per institutional contracted prices and daily dose as per indication or dose adjustment for hepatic or renal dysfunction.

## Conclusion

This study presents the NBL use and financial impact in the largest identified cohort of inpatients undergoing PST in an urban, community, and non-teaching medical center. Results highlight significant avoidance of NBL DOT. Associated cost avoidance contributes to the sustainability and longevity of an allergy management program.
